# Intermittent Hepatic Inflow Occlusion During Partial Hepatectomy for Hepatocellular Carcinoma Does Not Shorten Overall Survival or Increase the Likelihood of Tumor Recurrence

**DOI:** 10.1097/MD.0000000000000288

**Published:** 2014-12-02

**Authors:** Jiwei Huang, Wei Tang, Roberto Hernandez-Alejandro, Kimberly A. Bertens, Hong Wu, Mingheng Liao, Jiaxin Li, Yong Zeng

**Affiliations:** From the Department of Liver Surgery, Division of Liver Transplantation, West China Hospital, Sichuan University, Chengdu, China (JH, HW, ML, JL, YZ); Department of Hepato-Biliary-Pancreatic Surgery, University of Tokyo Hospital, University of Tokyo, Tokyo, Japan (WT); Department of Hepato-Biliary-Pancreatic Surgery, London Health Sciences Centre, Western University, London, Canada (RHA, KAB)

## Abstract

**Aim:** To investigate whether the long-term outcomes of hepatocellular carcinoma (HCC) was adversely impacted by intermittent hepatic inflow occlusion (HIO) during hepatic resection.

**Methods:** 1549 HCC patients who underwent hepatic resection between 1998 and 2008 were identified from a prospectively maintained database. Intermittent HIO was performed in 931 patients (HIO group); of which 712 patients had a Pringle maneuver as the mechanism for occlusion (PM group), and 219 patients had selective hemi-hepatic occlusion (SO group). There were 618 patients that underwent partial hepatectomy without occlusion (occlusion-free, OF group).

**Results:** The 1-, 3-, and 5- year overall survival (OS) rates were 79%, 59%, and 42% in the HIO group, and 83%, 53%, and 35% in the OF group, respectively. The corresponding recurrence free survival (RFS) rates were 68%, 39%, and 22% in the HIO group, and 74%, 41%, and 18% in the OF group, respectively. There was no significant difference between the 2 groups in OS or RFS (*P* = 0.325 and *P* = 0.416). Subgroup analysis showed patients with blood loss over 3000 mL and those requiring transfusion suffered significantly shorter OS and RFS. Blood loss over 3000 mL and blood transfusion were independent risk factors to OS and RFS.

**Conclusions:** The application of intermittent HIO (PM and SO) during hepatic resection did not adversely impact either OS or RFS in patients with HCC. Intermittent HIO is still a valuable tool in hepatic resection, because high intraoperative blood loss resulting in transfusion is associated with a reduction in both OS and RFS.

## INTRODUCTION

Although liver transplantation and local ablation techniques have achieved remarkable progress; surgical resection remains an important modality for treatment of hepatocellular carcinoma (HCC).^1–5^ Efforts to minimize intraoperative blood loss have been the primary focus throughout the history of liver surgery.^6^ Techniques to occlude the hepatic vascular inflow are widely-adapted, efficient maneuvers to manage intraoperative hemorrhage during parenchymal transection.^7–9^ The Pringle maneuver (PM), first described more than a century ago for hepatic trauma,^10^ is the most commonly utilized method to achieve inflow occlusion. Intermittent PM is generally felt to be the simplest and safest way to curtail severe intraoperative hemorrhage, however, the potential for ischemia-reperfusion (I/R) injury to the liver parenchyma and its resultant impact has been long debated.^11–15^

The I/R injury that occurs as a result of intermittent PM appears to be temporary and reversible, thus intermittent PM is considered an acceptable maneuver in hepatic resection, and can be used even in graft harvesting for living donor liver transplantation.^7,8,16–18^ Nonetheless, little attention has been paid to its impact on the long-term oncological outcomes. Recently, it was reported in multiple rodent studies that I/R injury may negatively impact the oncological outcomes.^19–32^ If a similar effect can be demonstrated in humans, the application of intermittent PM or other intermittent HIO techniques in the setting of oncologic resection would need to be cautioned. Thus, an investigation into the impact of intermittent HIO on the long-term outcome in patients undergoing hepatic resection for malignancy is urgently required.

Our hypothesis is that a correlation exists between the application of intermittent intraoperative HIO on both overall survival (OS) and recurrence free survival (RFS) in patients undergoing hepatic resection for HCC. To test this hypothesis, we conducted a cohort study in a population of HCC patients undergoing resection, comparing the long-term outcomes between those received intermittent HIO and those without inflow occlusion (OF).

## METHODS

### Study Population

The study was performed according to the guidelines of the Helsinki Declaration and approved by the institutional review board of West China Hospital. Prior to operation, written informed consent was signed by the enrolled patient or substitute decision maker.

From March 1998 to March 2008, 2819 consecutive HCC patients underwent partial hepatectomy with curative intent in the West China Hospital.^2^ A total of 1549 patients were identified from a prospectively maintained database. They were included for our study conforming to the following criteria: patients who were treated with hepatic resection as a first-line treatment modality (patients who received multiple resections were only included for initial operation); a diagnosis of HCC confirmed by pathology; Child-Pugh Class A preoperative liver function; without detectable extrahepatic metastasis; without previous or simultaneous malignancies; patients in which a R0 resection was achieved. Exclusion criteria were: patients who were with incomplete or vague medical reports were excluded.

### Definitions and Surgical Procedure

Pre-conditioning maneuver was not applied in either type of occlusion.

*The intermittent Pringle maneuver*: the hepatoduodenal ligament was routinely clamped en masse for no more than 15 minutes every period, and then released for a 5-minute interval.

*The intermittent selective hemi-hepatic occlusion (SO)*: was performed after dissection of the hepatoduodenal ligament. Following the removal of the gallbladder, the left, right anterior and right posterior branches of the hepatic artery and portal vein were identified and encircled separately. The corresponding arterial and portal venous branches were clamped during liver parenchymal transection. The clamping routinely lasted less than 30 minutes every period, and then released for a 5-minute interval.

*Major hepatic resection*: defined as removal of 3 or more hepatic segments according to the Couinaud classification.

*Anatomical resection:* since we did not apply dye-injection techniques, anatomical subsegmentectomy^33^ was not performed.

*Blood transfusio*n: for the purpose of this study, only allogeneic transfusion was considered.

*Postoperative mortality*: defined as death within 30 days of operation.

*Postoperative complications:* were defined as morbidity within 30 days of operation and classified according to the accordion severity grading system of postoperative complications.^34^

*Early recurrence:* defined as tumor recurrence within 2 years after operation^35^

*Surgical technique:* parenchymal transection was performed using a variety of instruments including; cavitron ultrasonic aspiration (CUSA, Valleylab Corp. Somerville, NJ,US.), water dissection (Jet2, Erbe Corp., Tuebingen, Germany), Harmonic scalpel (Johnson & Johnson Corp. Princeton, NJ.US) and Ligasure (Valleylab Corp. Somerville, NJ,US.) according to the operating surgeon's preference. However, clamp-crashing was the most frequently used method patients who underwent inflow occlusion.^36^

### Follow-Up

We routinely execute a three-month-interval follow-up program^1,2,37^ for all HCC patients discharged from hospital post intervention for curative intent.

### Statistical Analysis

Differences between the 2 groups were analyzed by the unpaired *t* test for continuous variables, and the χ^2^ test or continuity correction method for categorical variables. The OS curves and RFS curves were generated by the Kaplan–Meier method and compared by log-rank test. The data of patients who were lost in follow-up were censored. The relative prognostic significance of the variables in predicting OS and RFS were assessed by univariate and multivariate *Cox* proportional hazards regression models. All variables with a *P*-value < 0.05 by univariate comparison were subjected to the multivariate analysis. Results of the multivariate analysis were presented as relative risk (RR) with a corresponding 95% confidence interval (CI). All statistical tests were 2-sided, and a significant difference was considered when *P* < 0.05. The statistical analyses of the data were performed using the SPSS 17.0 statistical software (SPSS Company, Chicago, IL).

## RESULTS

The study population consisted of 1549 patients. Of this, 931 patients underwent partial hepatectomy with intermittent HIO (HIO group); whereas 618 patients received occlusion-free operations (OF group). Within the HIO group, the majority of patients (712) were managed with intermittent PM (PM group), and the remainder (219 patients) were treated with intermittent SO (SO group). The mean clamping time was 47.4 ± 38.7 minutes (3–208 min) for the PM group and 53.1 ± 33.5 minutes (19–112 min) for the SO group.

### Demographic and Clinicopathologic Analysis

The preoperative demographic variables of the 1549 patients were analyzed, and the details are shown in Table 1. The OF and the HIO group were comparable on the majority of the variables examined, excluding gender, proportion with liver cirrhosis, and mean serum alpha fetal protein (AFP).

**TABLE 1:**
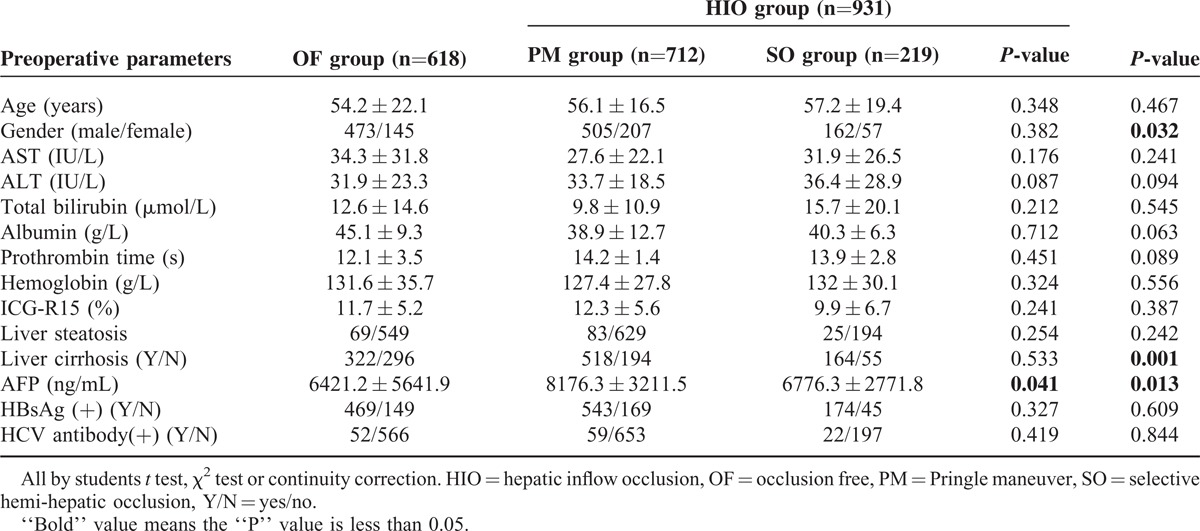
Preoperative Demographic Features of the Study Population

The intraoperative and postoperative variables were shown in Table 2. Intuitively, the PM group had a larger mean dominant tumor size, more major resections and anatomical resections than the SO group and OF group, whereas the mean operation time was shorter in the PM group. With regards to the mean estimated blood loss and necessity for transfusion, the PM group and the SO group were comparable; however the OF group had a significantly higher mean estimated blood loss and significantly more patients required blood transfusion.

**TABLE 2:**
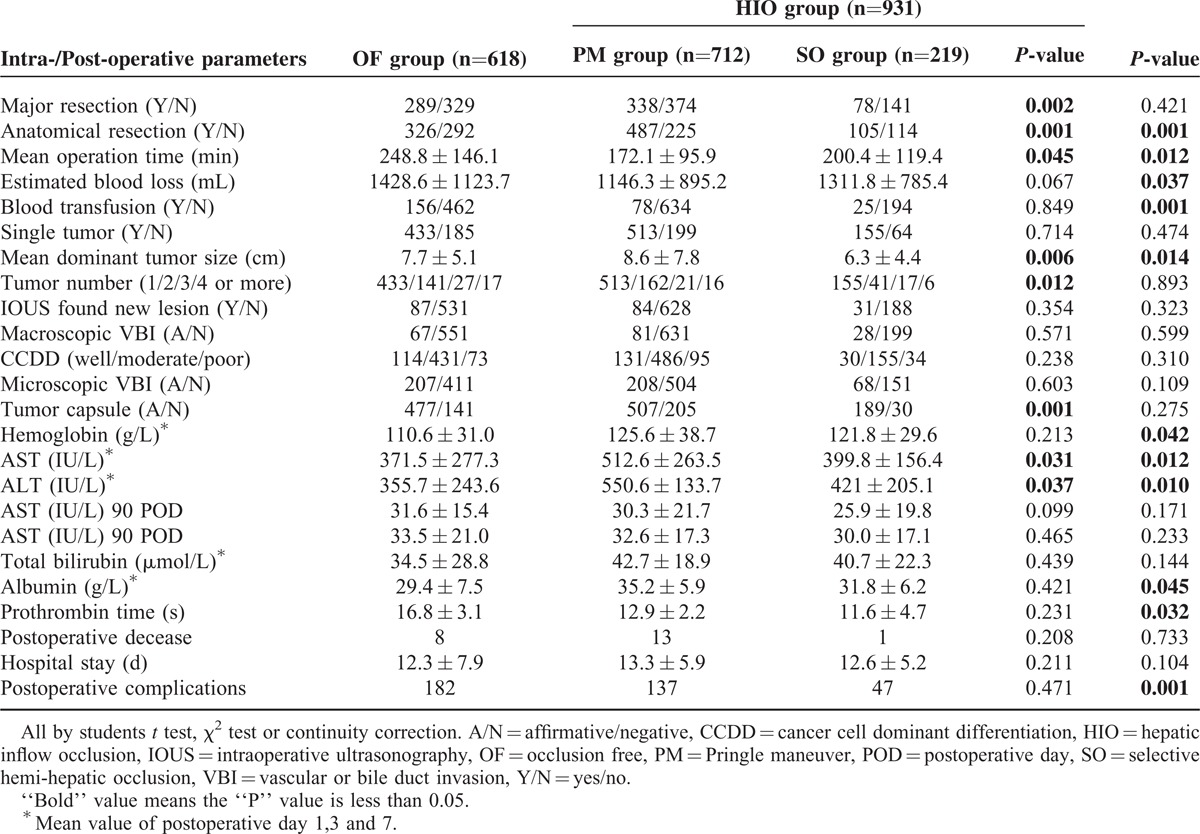
Intra-/Postoperative Features of the Study Population

Post-operative mortality was 1.3% in the OF group and 1.5% in the HIO group (*P* = 0.733). The likelihood of a postoperative complication was significantly higher in the OF group (29.4% vs 19.8%, *P* = 0.001). The details are shown in Table 3.

**TABLE 3:**
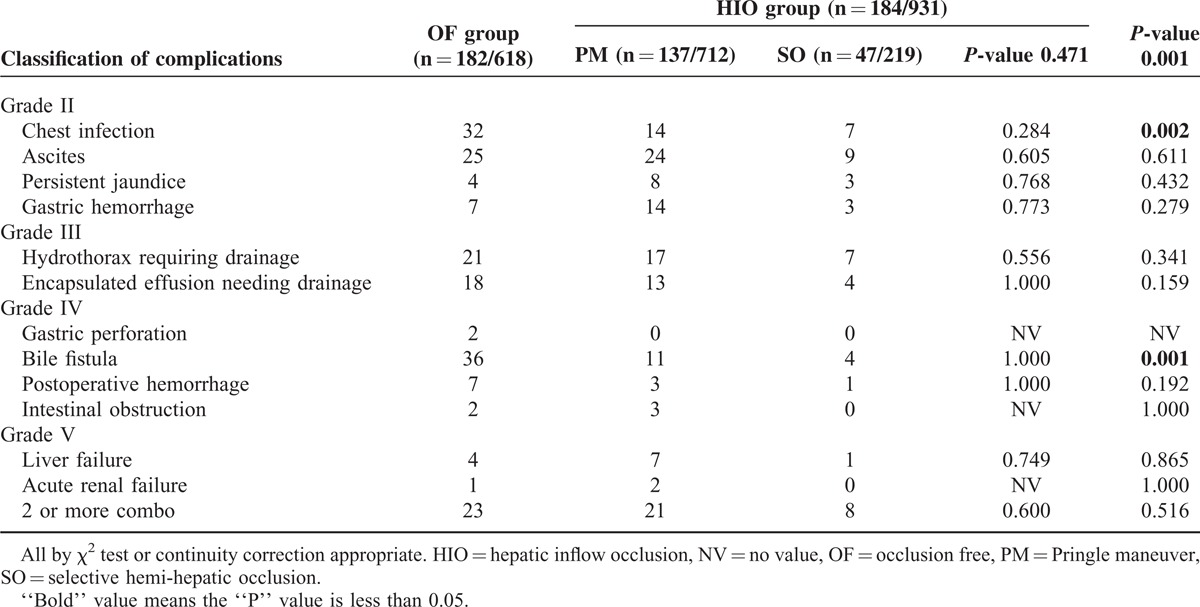
Data of Postoperative Complications of the Study Population

### Survival

The mean follow-up time was 68.4 ± 57.8 months (1–168 months). In the OF group, 385 patients died (62%), and 21 were lost to follow-up (3% censored). The most common cause of death in the OF group was cancer recurrence (77%), liver failure (6%), and upper gastrointestinal hemorrhage (4%). In the HIO group, 547 patients were deceased (59%) while 47 patients were lost to follow-up during the study period (5% censored). Again the common causes of death were cancer recurrence (86%), liver failure (3%), and upper gastrointestinal hemorrhage (2%).

**FIGURE 1:**
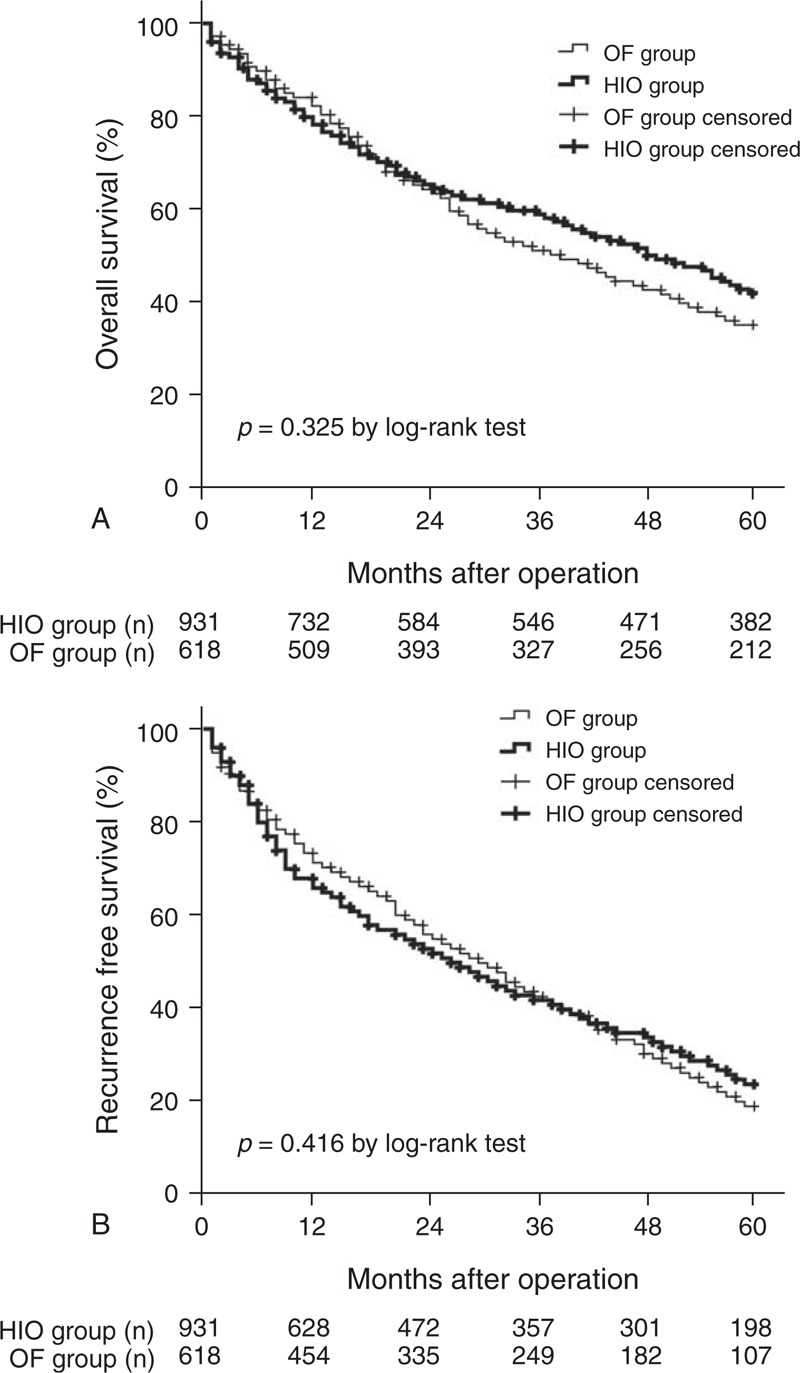
(A) The comparison of overall survival of the HIO group and OF group. (B) The comparison of recurrence free survival of the HIO group and OF group.

The 1-, 3- and 5-year OS rates were 79%, 59%, and 42% in the HIO group, and 83%, 53%, and 35% in the OF group, respectively. There was no significant difference between the 2 groups in OS (*P* = 0.325 by log-rank test, Figure 1A). The corresponding RFS rates were 68%, 39%, and 22% in the HIO group, and 74%, 41%, and 18% in the OF group, respectively. Again, there was no significant difference between the 2 groups in RFS (*P* = 0.416 by log-rank test, Figure 1B).

### Recurrence

At the endpoint of this study, recurrence was documented in 436 (71%) patients in the OF group, and 633 (68%) patients in the HIO group. There was no significant difference found in risk of recurrence between the 2 groups (*P* = 0.286). Furthermore, there was no significant difference in early recurrence (recurrence < 2 years)(*P* = 0.113) between the OF group (304 patients) and the HIO group (412 patients). With regards to the site of recurrence, intrahepatic recurrence was the most common site and occurred in 273 (63%) patients in the OF group, 370 (58%) patients in the HIO group. Extrahepatic recurrence was diagnosed in 163 patients (37%) of the OF group, 262 patients (41%) in the HIO group. Only 1 patient in the HIO group was found to have intrahepatic recurrence and lung dissemination simultaneously. There was no significant difference found in recurrence location (*P* = 0.182). In addition, there were no significant differences found in total recurrence, early recurrence and recurrence location when comparing the PM and SO groups (*P* = 0.273, *P* = 0.154, *P* = 0.151).

### Subgroup Analysis

A subgroup survival analysis was carried out in 4 different subgroups as follows; *a*. the PM group vs SO group; *b*. patients with single tumor less than 5 cm from the OF group vs HIO group; *c*. OF group patients with liver cirrhosis vs HIO group and *d*. patients undergoing major resections with no inflow occlusion vs HIO group. There was no significant difference found in any of the subgroup analyses for OS and RFS. Details are shown in Table 4.

**TABLE 4:**
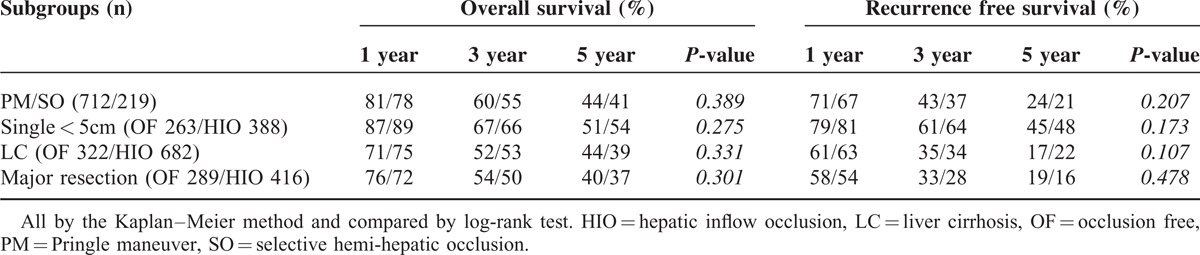
Overall Survival and Recurrence Free Survival Analysis of the Subgroups

*Intraoperative blood loss ≥ 3000 mL:* In the study, there were 63 patients that encountered an estimated intraoperative blood loss of ≥ 3000 mL. Among these patients, the mean estimated blood loss was 4515 mL (3150–9750 mL), and all received blood transfusion. The mean size of the dominant tumor was 7.6 cm (4.5–16.8 cm). All 63 patients underwent a major resection. Additionally, 89% were cirrhotic and 62 of 63 patients had pathological evidence of macroscopic vascular or bile duct invasion. The perioperative mortality in this subgroup was 23.8%. These 63 patients were divided into 2 groups by the number of lesions, ie, 39 patients with single lesion (massive hemorrhage, MH group 1) and 24 with 2 or more lesions (MH group 2).

A matched analysis was performed with 128 patients who were selected from the rest of the study pool of patients with blood loss less than 3000 mL and without blood transfusion. They were matched to the 63 patients above by the following variables: major hepatectomy, mean dominant tumor diameter larger than 7.8 cm, liver cirrhosis, and macroscopic vascular or bile duct invasion. The mean estimated blood loss of the contrast group was 644 mL (250–1000 mL). The mean dominant tumor size was 8.5 cm (8.0–17.5 cm). Eighty-one patients had a single lesion (contrast group 1), and 47 patients had multiple lesions (contrast group 2). The perioperative mortality in these contrast subgroups was 0.8%.

**FIGURE 2:**
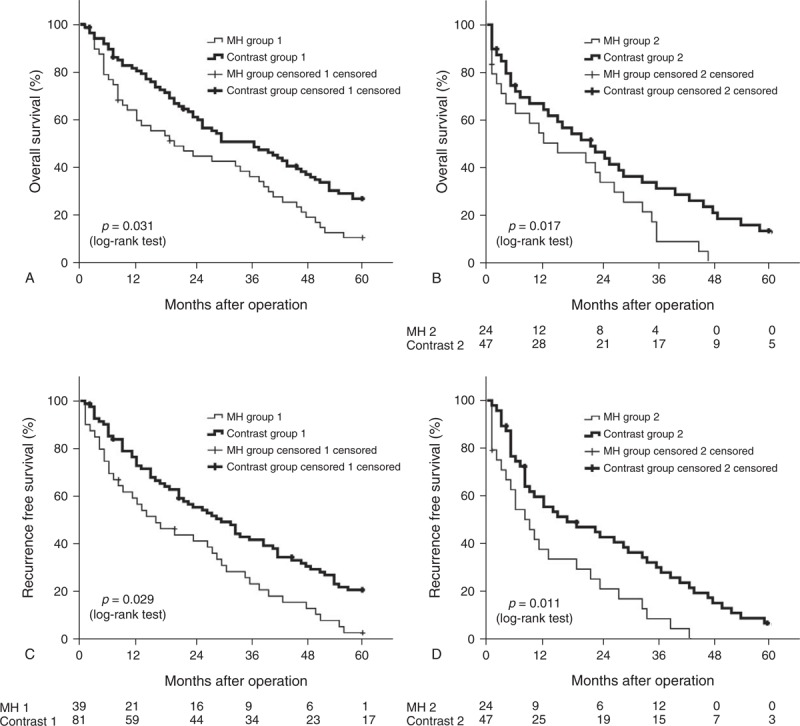
(A) The comparison of overall survival of the MH group 1 and contrast group 1 (B) The comparison of overall survival of the MH group 2 and contrast group 2 (C) The comparison of recurrence free survival of the MH group 1 and contrast group 1 (D) The comparison of recurrence free survival of the MH group 2 and contrast group 2.

The 1-, 3- and 5-year OS rates were 68%, 35%, and 12% in the MH group 1, and 79%, 48%, and 14% in the contrast group 1, respectively. The contrast group 1 had a significantly better OS than the MH group 1 (*P* = 0.031 by log-rank test, Figure 2A). The 1-, 3- and 5-year OS rates were 53%, 17%, and 0% in the MH group 2, and 63%, 39%, and 11% in the contrast group 2, respectively. The OS of the contrast group 2 was significantly better than the MH group 2 as well (*P* = 0.017 by log-rank test, Figure 2B).

The 1-, 3- and 5-year RFS rates were 57%, 24%, and 2% in the MH group 1, and 71%, 40%, and 18% in the contrast group 1, respectively. The RFS was significantly better in the contrast group 1 compared to the MH group 1 (*P* = 0.029 by log-rank test, Figure 2C). The 1-, 3- and 5-year RF rates were 38%, 8%, and 0% in the MH group 2, and 56%, 33%, and 6% in the contrast group 2, respectively. The RFS of the contrast group 2 was significantly better than the MH group 2 (*P* = 0.011 by log-rank test, Figure 2D).

### The Univariate and Multivariate Analyses for OS and RFS

The univariate and multivariate analyses for OS and RFS are shown in Table 5. In the univariate analysis, 18 of 26 included variables were found to be predictive to OS. However, in the multivariate analysis, only 11 variables were independently associated with OS. The corresponding RRs to OS were: 1.51 for age over 65 years, 2.23 for HCV infection, 2.67 for liver cirrhosis, 2.98 for serum AFP over 400 ng/L, 2.17 for major resection, 1.51 for non-anatomical resection, 4.68 for blood loss over 3000 mL, 3.12 for blood transfusion, 5.74 for macroscopic and 1.62 for microscopic vascular or bile duct invasion, and 1.89 for cancer cell dominant differentiation.

**TABLE 5:**
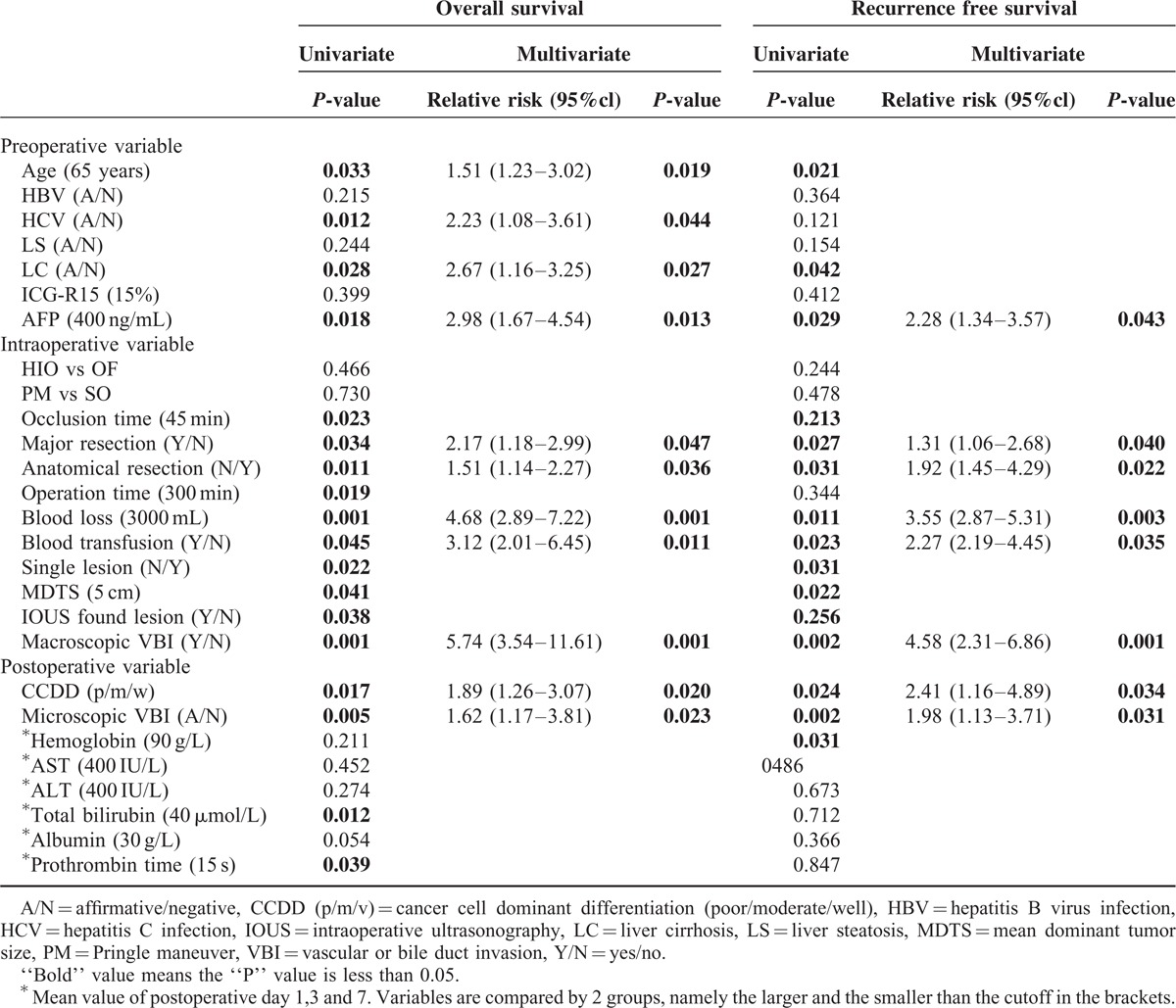
Univarite and Multivariate Analysis of the Relative Risk for OS and RFS

In univariate analysis, 16 of the 26 variables were found to be associated with RFS. However, only 8 variables were seen to be independently associated with RFS in the multivariate analysis. Their RRs to RFS were as follows: 2.28 for serum AFP over 400 ng/L, 1.31 for major resection, 1.92 for non-anatomical resection, 3.55 for blood loss over 3000 mL, 2.27 for blood transfusion, 4.58 for macroscopic and 1.98 for microscopic vascular or bile duct invasion, and 2.41 for cancer cell dominant differentiation.

Regardless of technique, there was no association between HIO and either RFS or OS, in univariate or multivariate analysis.

## DISCUSSION

Our data demonstrated that surgeons preferred to perform PM in the complicated cases (liver cirrhosis, major and anatomical resection, and large or multiple lesions), and seemed to achieve more optimistic perioperative results (shorter operation time, lower blood loss, lower rate of blood transfusion, and decreased morbidity). This is accordance with the benefits that have already demonstrated before that improved surgical visibility with decreased bleeding in the setting of HIO, and it could be expected to result in better short-term operative results.^9,10,38–40^

Of note, the HIO group in this study carries with some surrogate markers for more advanced tumor features, more cirrhotic liver background and more extensive surgery than the OF group, which could reasonably result in a worse long-term outcome. On the contrary, there were no significant differences in OS, RFS (Figure 1) or patterns of recurrence between the 2 groups. Conceptually, if the baseline parameters are more equivalent between the 2 groups; the HIO group might possibly achieve better oncological outcomes than the OF group. Interestingly, this inherent selection biases that arise because of the retrospective nature of our study would actually serve to make it more likely that the HIO group would have shorter OS and RFS. On the other hand, the diminished blood loss and lower rate of blood transfusion in the HIO group may contribute to the equivalent survival. In this study, we note that patients with severe blood loss had high perioperative mortality, and inferior OS and RFS. Not surprisingly, in the univarite and multivariate analysis, severe blood loss and blood transfusion were predictive of a lower OS and RFS. Our results are concordant with previously published studies, demonstrating that a reduction in intraoperative blood loss has a favorable outcome on both short- and long-term outcomes.^41–45^

In addition in both univariate and multivariate analysis, HIO was not predictive of either OS or RFS (Table 5). Similar results in patients with colorectal liver metastases have been reported in the literatures including several retrospective cohort studies, a randomized control trial and a meta-analysis.^46–50^ Nonetheless, the mechanisms of dissemination are completely different between HCC and colorectal liver metastases. The evidence in patients with HCC is lacking on this topic. To date, only 2 retrospective studies with small sample sizes exist reporting conflicting results.^51,52^ Thus, the results of our study may have its value. Of note, by reviewing animal experiments that initially demonstrated the link between HIO and poor oncological results, one can see that the diversity of experimental procedures, and even species diversity, vastly alters the results.^19–32^

Two recently published meta-analyses showed no statistically significant differences in mortality or morbidity between the different vascular occlusion techniques.^53,54^ Similar results were found in our study. We too found that the technique of inflow occlusion (PM vs SO) did not significantly impact OS or RFS. Thus, we would argue that the PM should be the favored approach due to its simplicity and atraumatic nature.

Subgroup analysis was also performed on patients with early stage tumors (single tumor less than 5 cm), patients with liver cirrhosis, and patients undergoing major resections. No significant differences were found between the HIO group and the OF group, which demonstrates our results exhibit external validity and as such are generalizable to various populations.

This study clearly has limitations and shortcomings as a result of its retrospective cohort design: firstly, 366 of 2819 cases were excluded because of incomplete or vague medical records; secondly, the study time was over 10 years, and there were 23 operative surgeons involved in this study; thirdly, there was no specific algorithm for applying HIO during hepatic resection, thus whether HIO (selective or not) was applied was at the operating surgeon's discretion. This may introduce some selection biases. However, to our knowledge, this study represents the largest study to date on the long-term outcomes following HIO in a population of patients undergoing hepatic resection with curative intent for HCC. A prospective trial has yet to be published.

## CONCLUSION

Intermittent hepatic inflow occlusion during hepatic resection has no adverse impact on the OS or the RFS for patients with HCC. Furthermore, there is no significant difference in either short- or long-term oncologic outcomes in patients receiving the PM as compared to selective hemi-hepatic occlusion. The ease and relatively atraumatic nature of the PM make it preferable to selective hepatic occlusion. Intermittent hepatic inflow occlusion could be utilized liberally because severe hemorrhage and the necessity for blood transfusion are independently associated with both a decreased OS and RFS.
